# Rac1 Activation Caused by Membrane Translocation of a Guanine Nucleotide Exchange Factor in Akt2-Mediated Insulin Signaling in Mouse Skeletal Muscle

**DOI:** 10.1371/journal.pone.0155292

**Published:** 2016-05-10

**Authors:** Nobuyuki Takenaka, Yuma Nihata, Takaya Satoh

**Affiliations:** Laboratory of Cell Biology, Department of Biological Science, Graduate School of Science, Osaka Prefecture University, Sakai, Osaka, Japan; University of Texas Health Science Center at Houston, UNITED STATES

## Abstract

Insulin-stimulated glucose uptake in skeletal muscle is mediated by the glucose transporter GLUT4, which is translocated to the plasma membrane following insulin stimulation. Several lines of evidence suggested that the protein kinase Akt2 plays a key role in this insulin action. The small GTPase Rac1 has also been implicated as a regulator of insulin-stimulated GLUT4 translocation, acting downstream of Akt2. However, the mechanisms whereby Akt2 regulates Rac1 activity remain obscure. The guanine nucleotide exchange factor FLJ00068 has been identified as a direct regulator of Rac1 in Akt2-mediated signaling, but its characterization was performed mostly in cultured myoblasts. Here, we provide *in vivo* evidence that FLJ00068 indeed acts downstream of Akt2 as a Rac1 regulator by using mouse skeletal muscle. Small interfering RNA knockdown of FLJ00068 markedly diminished GLUT4 translocation to the sarcolemma following insulin administration or ectopic expression of a constitutively activated mutant of either phosphoinositide 3-kinase or Akt2. Additionally, insulin and these constitutively activated mutants caused the activation of Rac1 as shown by immunofluorescent microscopy using a polypeptide probe specific to activated Rac1 in isolated gastrocnemius muscle fibers and frozen sections of gastrocnemius muscle. This Rac1 activation was also abrogated by FLJ00068 knockdown. Furthermore, we observed translocation of FLJ00068 to the cell periphery following insulin stimulation in cultured myoblasts. Localization of FLJ00068 in the plasma membrane in insulin-stimulated, but not unstimulated, myoblasts and mouse gastrocnemius muscle was further affirmed by subcellular fractionation and subsequent immunoblotting. Collectively, these results strongly support a critical role of FLJ00068 in Akt2-mediated Rac1 activation in mouse skeletal muscle insulin signaling.

## Introduction

Glucose is transported into the cell in response to insulin by the glucose transporter GLUT4 in skeletal muscle and adipose tissue [1–3]. In unstimulated cells, GLUT4 is sequestered in specific intracellular compartments termed GLUT4 storage vesicles. Induction of glucose uptake by insulin occurs through the redistribution of GLUT4 from GLUT4 storage vesicles to the plasma membrane. Various events that occur during GLUT4 vesicle exocytosis are thought to be enhanced via signaling networks downstream of the insulin receptor. Signaling mechanisms for the regulation of GLUT4 vesicle transport involve the activation of a kinase cascade composed of phosphoinositide 3-kinase (PI3K) and protein kinases such as PDK1 and Akt2. Activated Akt2 in turn induces phosphorylation of its protein substrates, leading to GLUT4 translocation to the plasma membrane.

The Akt substrate of 160 kDa (AS160, also termed TBC1D4) [4] is one of the best-characterized targets of Akt2 that are involved in insulin-dependent glucose uptake. AS160 is a GTPase-activating protein for Rab GTPases that regulate GLUT4 vesicle trafficking, including Rab8A, Rab10, and Rab13 [4, 5]. Phosphorylation of AS160 by Akt2 attenuates its GTPase-activating protein activity, leading to the activation of the above Rab proteins [6, 7]. TBC1D1, a close relative of AS160, is another substrate of Akt2, regulating Rab protein activity and GLUT4 translocation [8]. In addition to the above Rab GTPase-activating proteins, diverse Akt substrates have been implicated in GLUT4 translocation [9–14]. However, the detailed mechanisms for the action of Akt2 remain only partly understood.

We and others have recently demonstrated that the Rho family small GTPase Rac1 plays a pivotal role in the regulation of GLUT4 translocation in skeletal muscle [15–22]. Indeed, impaired glucose tolerance and higher plasma insulin concentrations after intraperitoneal glucose injection were observed in muscle-specific *rac1* knockout (m-*rac1*-KO) mice [20]. However, detailed mechanisms whereby Rac1 is activated in response to insulin remain incompletely understood, and therefore, we attempted to clarify these mechanisms by the use of cultured myoblasts and m-*rac1*-KO mice. The activation of Rac1 was observed when a myristoylated form of the PI3K catalytic subunit p110α (Myr-p110α) or myristoylated Akt2 (Myr-Akt2), as a constitutively activated mutant, was ectopically expressed in L6 myoblasts and mouse gastrocnemius muscle fibers [23–25]. Moreover, constitutively activated forms of PI3K and Akt2 stimulated GLUT4 translocation in wild-type, but not in m-*rac1*-KO, mouse gastrocnemius muscle fibers [24]. Taken together, these results strongly support a model in which Rac1 is regulated downstream of Akt2 in insulin signaling that directs GLUT4 translocation in skeletal muscle. On the other hand, another model in which Rac1 is regulated downstream of PI3K, but not Akt2, and Akt2 and Rac1 are responsible for GLUT4 vesicle exocytosis and cytoskeletal rearrangements, respectively is also proposed [15, 26, 27].

The GTP/GDP state of Rac1 is modulated by specific GEFs in response to upstream signals. Through screening of GEFs for Rac1 that are expressed in skeletal muscle, we identified the Dbl family member FLJ00068 (also termed PLEKHG4 or puratrophin-1) as a guanine nucleotide exchange factor (GEF) that is responsible for Rac1 activation in insulin signaling in L6 myoblasts [21]. Furthermore, Rac1 activation and GLUT4 translocation following ectopic expression of either constitutively activated PI3K or constitutively activated Akt2 were totally diminished by small interfering RNA (siRNA)-mediated knockdown of FLJ00068 in L6 cells [24, 28]. On the other hand, a constitutively activated mutant of FLJ00068 actually caused GLUT4 translocation in a Rac1-dependent manner [28]. Although these results in L6 myoblasts suggest a critical role of FLJ00068 in the regulation of Rac1 downstream of Akt2 in skeletal muscle insulin signaling, validation of this model in mouse skeletal muscle remains incomplete.

We have not yet examined the effect of depletion of FLJ00068 on Rac1 activation and GLUT4 translocation in mouse skeletal muscle because FLJ00068 gene knockout mice are currently not available in our laboratory. However, our recent progress in siRNA-mediated knockdown and *in situ* detection of the activation of small GTPases in mouse skeletal muscle [25, 29] enabled us to test the involvement of FLJ00068 in Akt2-dependent activation of Rac1. In this study, we aim to provide *in vivo* evidence for the role of the GEF FLJ00068 in Akt2-dependent activation of Rac1 in mouse skeletal muscle. Moreover, we describe a novel and convenient method to detect Rac1 activation in a frozen section of mouse gastrocnemius muscle by immunofluorescent microscopy, which reinforces the results obtained from the analysis in isolated muscle fibers.

## Materials and Methods

### Materials

A rat monoclonal antibody against the hemagglutinin (HA) epitope tag (11 867 423 001) was purchased from Roche Applied Science (Germany). A mouse monoclonal antibody against the Myc epitope tag (05–724) was purchased from Millipore (MA, USA). A goat polyclonal antibody against the V5 epitope tag (A190-119A) was purchased from Bethyl (TX, USA). A rabbit polyclonal antibody against FLJ00068 (ab137898) was purchased from Abcam (UK). A mouse monoclonal antibody against Rac1 (61065) was purchased from BD bioscience (CA, USA). A rabbit polyclonal antibody against p-PAK1(Thr423) (sc-12925) was purchased from Santa Cruz Biotechnology (CA, USA). Mouse monoclonal antibodies against α-tubulin (T9026) and Na^+^/K^+^-ATPase (A-277) were purchased from SIGMA-Aldrich (MO, USA). Antibodies against goat IgG, mouse IgG, rabbit IgG, and rat IgG conjugated with CF™ 405/488/543/647 were purchased from Biotium (CA, USA). Insulin was purchased from Eli Lilly (IN, USA). Two siRNA duplexes against mouse FLJ00068, #1 (Genosys (MO, USA), Plekhg4_3514; 5’-GCAACUAUGGCCACACCU-3’) and #2 (Genosys, Plekhg4_3515; 5’-CGAUUACAGGUCUGCAGUA-3’), and a mixture of non-targeting control (NC) siRNA duplexes (Dharmacon (CO, USA), D-001206-13; Duplex 1, 5'-AUGAACGUGAAUUGCUCAAUU-3'; Duplex 2, 5'-UAAGGCUAUGAAGAGAUACUU-3'; Duplex 3, 5'-AUGUAUUGGCCUGUAUUAGUU-3'; and Duplex 4, 5'-UAGCGACUAAACACAUCAAUU-3') were commercially obtained.

### Cell culture

The L6-GLUT4 cell line is derived from L6 rat myoblasts, and stably expresses the GLUT4 reporter GLUT4*myc*7-green fluorescent protein (GFP) [21, 23, 24, 28, 30]. L6-GLUT4 cells were cultivated in Dulbecco's modified Eagle's medium (08458–16, Nacalai (Japan)) supplemented with 10% (v/v) fetal bovine serum (Biowest (France)), 1 mM sodium pyruvate, 100 IU/ml penicillin, and 100 μg/ml streptomycin.

### Animal experiments

Adult male mice with the C57BL/6 genetic background were used in this study. This study was carried out in strict accordance with the recommendations in the Guidelines for Proper Conduct of Animal Experiments of the Science Council of Japan. The protocol was approved by the Committee on the Ethics of Animal Experiments of Osaka Prefecture University (Permit Number: 27–83). Surgery was performed under anesthesia by intraperitoneal injection of a solution of medetomidine, midazolam, and butorphanol as described below, and all efforts were made to minimize suffering.

### Reverse transcription-polymerase chain reaction (RT-PCR)

The total cellular RNA was isolated from L6-GLUT4 cells and gastrocnemius muscle and the brain of wild-type C57BL/6 mice using the TRIzol^®^ RNA isolation reagent (Life Technologies (CA, USA)) according to the manufacturer’s instructions. cDNAs were synthesized using the SuperScript III first-strand synthesis system for RT-PCR (Life Technologies) and then amplified using KOD FX neo (Toyobo (Japan)) and specific primers (Life Technologies) (5’-GGCAGTTGGTACGACAGGAT-3’ and 5’-CGAAGCGCAGTTTACTTTCC-3’ for FLJ00068, and 5’-CCCCTTCATTGACCTCAACTAC-3’ and 5’-ATGACCTTGCCCACAGCCTTGG-3’ for glyceraldehyde-3-phosphate dehydrogenase (GAPDH)) according to the manufacturer’s instructions. Genome sequences recognized by the above primers are identical between rat and mouse.

### Immunohistochemical staining

Gastrocnemius muscle of wild-type C57BL/6 mice was fixed in 40 mg/ml paraformaldehyde and frozen in OCT compound (Sakura Finetek (USA)). Frozen sections were immunostained with anti-FLJ00068 and fluoresceinated secondary antibodies. Images were obtained and analyzed using a confocal laser-scanning microscope (FV1200, Olympus (Japan)).

### Gene transfer into mouse skeletal muscle by electroporation

Plasmid DNAs and siRNAs were introduced into mouse gastrocnemius muscle by electroporation. Adult male C57BL/6 mice were anesthetized by intraperitoneal injection of a solution of medetomidine (0.3 mg/kg of body weight), midazolam (4.0 mg/kg of body weight), and butorphanol (5.0 mg/kg of body weight). A combination of expression vectors (pCAGGS-GLUT4*myc*7-GFP [22], pCAGGS-Myr-p110α-HA×3 [24], pCAGGS-Myr-Akt2-HA×3 [24], and pCAGGS-HA×2-FLJ68ΔN [28]) (80 μg in total) and, where indicated, siRNAs (NC, #1, or #2) (1.7 μg in total) were dissolved in 50 μl of 9 mg/ml NaCl, and injected longitudinally into gastrocnemius muscle with a 29-gauge needle. A pair of stainless steel electrode needles fixed 5 mm apart were then inserted into the muscle belly, and square wave electrical pulses (50 milliseconds) were applied three times (100 V, 90 V, and 81 V, respectively) at 100-millisecond intervals (for poring) using a pulse generator (NEPA21 Type II, Nepa Gene (Japan)). Subsequently, square wave electrical pulses (50 milliseconds) were applied three times (20 V, 12 V, and 7.2 V, respectively) at 100-millisecond intervals followed by three more pulses under the same conditions except that the polarity is opposite (for transfer).

### Isolation of gastrocnemius muscle fibers and GLUT4 reporter assay

A GLUT4 reporter containing GFP and exofacial Myc tags (GLUT4*myc*7-GFP) was described previously [31]. Mice were fasted for 16 hours and anesthetized by intraperitoneal injection of a solution of medetomidine (0.3 mg/kg of body weight), midazolam (4.0 mg/kg of body weight), and butorphanol (5.0 mg/kg of body weight) 5 days after gene transfer by electroporation. Insulin was then administered intravenously where indicated. Gastrocnemius muscle was excised from anesthetized mice and fixed with 40 mg/ml paraformaldehyde in phosphate-buffered saline (PBS) for 45 minutes. Individual muscle fibers were teased from fixed muscle with fine forceps under stereomicroscopy, and incubated in 10 mg/ml bovine serum albumin in PBS for more than 30 minutes. Thereafter, muscle fibers were treated with an anti-Myc tag antibody (for the detection of GLUT4*myc*7-GFP translocated to the sarcolemma) for 1 hour, and washed three times with PBS. Subsequently, muscle fibers were permeabilized with 0.1% (v/v) Triton X-100 in PBS for 10 minutes, washed three times with 0.1% (v/v) Tween 20 in PBS, and incubated in 0.1% (v/v) Tween 20 in PBS supplemented with Mouse Ig Blocking Reagent (Vector Laboratories (CA, USA)) for 1 hour. Muscle fibers were then treated with antibodies against FLJ00068 and the HA tag (for the detection of Myr-p110α and Myr-Akt2) overnight at 4°C, and washed three times with PBS. Anti-Myc, anti-FLJ00068, and anti-HA antibodies were subsequently detected with fluoresceinated secondary antibodies. Images were obtained and analyzed using a confocal laser-scanning microscope (FV1200, Olympus). Fluorescent intensities of surface GLUT4 (Myc) and total GLUT4 (GFP) in total GLUT4-positive areas were quantified using ImageJ software. The relative amount of GLUT4*myc*7-GFP translocated to the sarcolemma was estimated by the ratio of Myc and GFP fluorescent intensities (Myc/GFP). Values of 6 muscle fibers in total from 3 different mice under each condition were used for statistical analysis (Student's t test).

### Isolation of gastrocnemius muscle fibers and detection of activated Rac1

Mice were fasted for 16 hours and anesthetized by intraperitoneal injection of a solution of medetomidine (0.3 mg/kg of body weight), midazolam (4.0 mg/kg of body weight), and butorphanol (5.0 mg/kg of body weight) 5 days after gene transfer by electroporation. Insulin was then administered intravenously where indicated. Gastrocnemius muscle was excised from anesthetized mice and fixed with 40 mg/ml paraformaldehyde in PBS for 45 minutes on ice. Individual muscle fibers were teased from fixed muscle with fine forceps under stereomicroscopy on ice, and fixed in overlay assay buffer (50 mM Hepes-NaOH (pH 7.3), 150 mM NaCl, 20 mM MgCl_2_, and 0.05% (v/v) Tween 20) supplemented with 20 mg/ml paraformaldehyde on ice for 1 minute. Thereafter, muscle fibers were treated with a glutathione *S*-transferase (GST) fusion of the V5 epitope-tagged Rac1-binding domain of POSH (GST-POSH(251–489)-V5×3) or V5 epitope-tagged GST (GST-V5×3) (10 μg/ml; purified from *Escherichia coli* transformants as described in Ref. 21) in overlay assay buffer supplemented with 0.1% (v/v) Triton X-100 and 50 μg/ml bovine serum albumin on ice for 20 minutes. After washing three times with overlay assay buffer, muscle fibers were fixed again in overlay assay buffer supplemented with 20 mg/ml paraformaldehyde on ice for 5 minutes. Fixed muscle fibers were washed with PBS supplemented with 0.05% (v/v) Tween20 three times, and incubated with an antibody against the V5 tag (for the detection of GST-POSH(251–489)-V5×3 or GST-V5×3). Muscle fibers were counterstained with antibodies against Rac1, FLJ00068, and the HA tag (for the detection of FLJ68ΔN, Myr-p110α, and Myr-Akt2). Anti-V5, anti-Rac1, anti-FLJ00068, and anti-HA antibodies were subsequently detected with fluoresceinated secondary antibodies. Images were obtained and analyzed using a confocal laser-scanning microscope (FV1200, Olympus). Fluorescent intensities of Rac1·GTP (V5) and Rac1 in Rac1-positive areas were quantified using ImageJ software. The activity of Rac1 was estimated by the ratio of V5 and Rac1 fluorescent intensities (V5/Rac1). Values of 6 muscle fibers in total from 3 different mice under each condition were used for statistical analysis (Student's t test).

### Detection of activated Rac1 in frozen sections of gastrocnemius muscle

Mice were fasted for 16 hours and anesthetized by intraperitoneal injection of a solution of medetomidine (0.3 mg/kg of body weight), midazolam (4.0 mg/kg of body weight), and butorphanol (5.0 mg/kg of body weight) 5 days after gene transfer by electroporation. Insulin was then administered intravenously where indicated. Gastrocnemius muscle was excised from anesthetized mice, fixed in 40 mg/ml paraformaldehyde in PBS for 30 minutes on ice, and frozen in OCT compound. Frozen sections were fixed in overlay assay buffer supplemented with 20 mg/ml paraformaldehyde on ice for 1 minute and treated with GST-POSH(251–489)-V5×3 or GST-V5×3 (10 μg/ml) in overlay assay buffer supplemented with 0.1% (v/v) Triton X-100 and 50 μg/ml bovine serum albumin on ice for 45 minutes. After washing three times with overlay assay buffer, frozen sections were fixed again in overlay assay buffer supplemented with 20 mg/ml paraformaldehyde on ice for 5 minutes. Fixed frozen sections were washed with PBS supplemented with 0.05% (v/v) Tween20 three times, and incubated with an antibody against the V5 tag (for the detection of GST-POSH(251–489)-V5×3 or GST-V5×3). Frozen sections were counterstained with antibodies against Rac1, phosphorylated PAK1, FLJ00068, and the HA tag (for the detection of FLJ68ΔN, Myr-p110α, and Myr-Akt2). Anti-V5, anti-Rac1, anti-p-PAK1(Thr423), anti-FLJ00068, and anti-HA antibodies were subsequently detected with fluoresceinated secondary antibodies. Images were obtained and analyzed using a confocal laser-scanning microscope (FV1200, Olympus). Fluorescent intensities of Rac1·GTP (V5) and Rac1 in Rac1-positive areas were quantified using ImageJ software. The activity of Rac1 was estimated by the ratio of V5 and Rac1 fluorescent intensities (V5/Rac1). Values of 6 frozen sections in total from 3 different mice under each condition were used for statistical analysis (Student's t test).

### Immunoblot analysis

Proteins separated by SDS-polyacrylamide gel electrophoresis were transferred on to a 0.45-μm pore size polyvinylidene difluoride membrane (GE Healthcare (UK)). Membranes were incubated with primary and horseradish peroxidase-conjugated secondary antibodies. Specific proteins were visualized by Chemi-Lumi One Ultra (Nacalai). Images were captured, and densitometric analysis was carried out by using a chemiluminescence imaging system (Ez-Capture MG, Atto (Japan)).

### Subcellular fractionation of L6-GLUT4 cells

L6-GLUT4 cells were serum-starved for 3 hours and then stimulated with or without 100 nM insulin for 30 minutes. Cells were washed with cold PBS, gently scraped into cold homogenization buffer (20 mM Hepes-NaOH (pH 7.3), 250 mM sucrose, 5 mM NaN_3_, 2 mM EGTA, and protease inhibitor cocktail (Nacalai), and homogenized on ice using a Teflon pestle homogenizer (20 strokes) (AS one (Japan)). The homogenate was centrifuged at 760×*g* for 5 minutes at 4°C to remove nuclei and unbroken cells. The supernatant was further centrifuged at 32,000×*g* for 1 hour at 4°C, yielding a pellet of the crude plasma membrane fraction and a supernatant of the cytosol fraction. The pellet was resuspended in cold homogenization buffer.

### Subcellular fractionation of mouse gastrocnemius muscle

C57BL/6 mice were fasted for 16 hours and anesthetized by intraperitoneal injection of a solution of medetomidine (0.3 mg/kg of body weight), midazolam (4.0 mg/kg of body weight), and butorphanol (5.0 mg/kg of body weight). Insulin was then administered intravenously where indicated. After 30 minutes, gastrocnemius muscle was excised from anesthetized mice, minced in a cold homogenization buffer, and homogenized on ice using a Teflon pestle homogenizer (50 strokes). Subcellular fractionation of gastrocnemius muscle was performed as described above for L6-GLUT4 cells.

## Results

**Fig 1:**
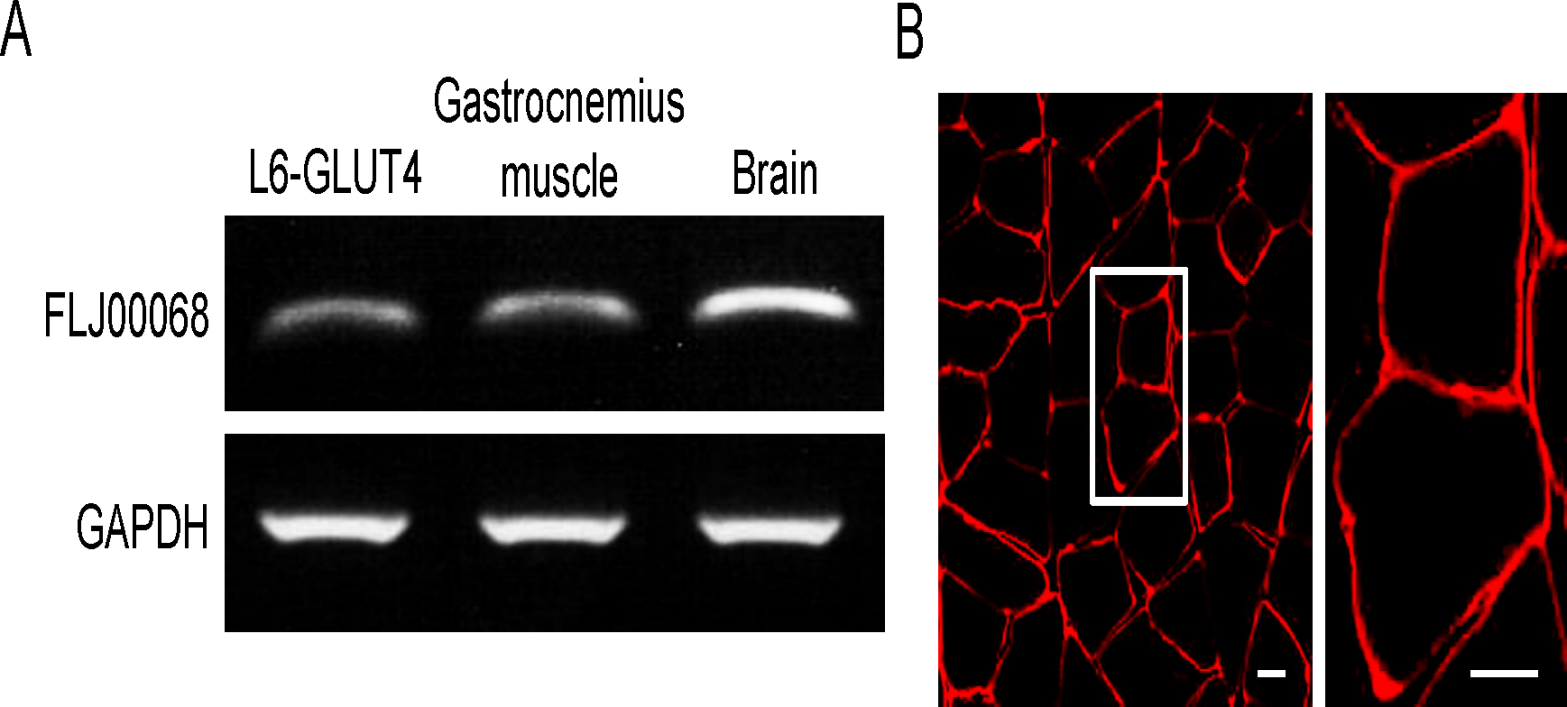
The expression of FLJ00068 in mouse gastrocnemius muscle. (A) Expression levels of FLJ00068 and GAPDH in L6-GLUT4 cells, mouse gastrocnemius muscle, and the mouse brain were assessed by RT-PCR. (B) FLJ00068 in the cross section of mouse gastrocnemius muscle was detected by immunohistochemical staining. The frozen section of mouse gastrocnemius muscle was stained with an anti-FLJ00068 antibody. The bundle of multiple muscle fibers can be seen in the left panel (low-magnification image). A couple of muscle fibers are shown in the right panel (high-magnification image of the boxed area in the left panel). Scale bar, 20 μm.

**Fig 2:**
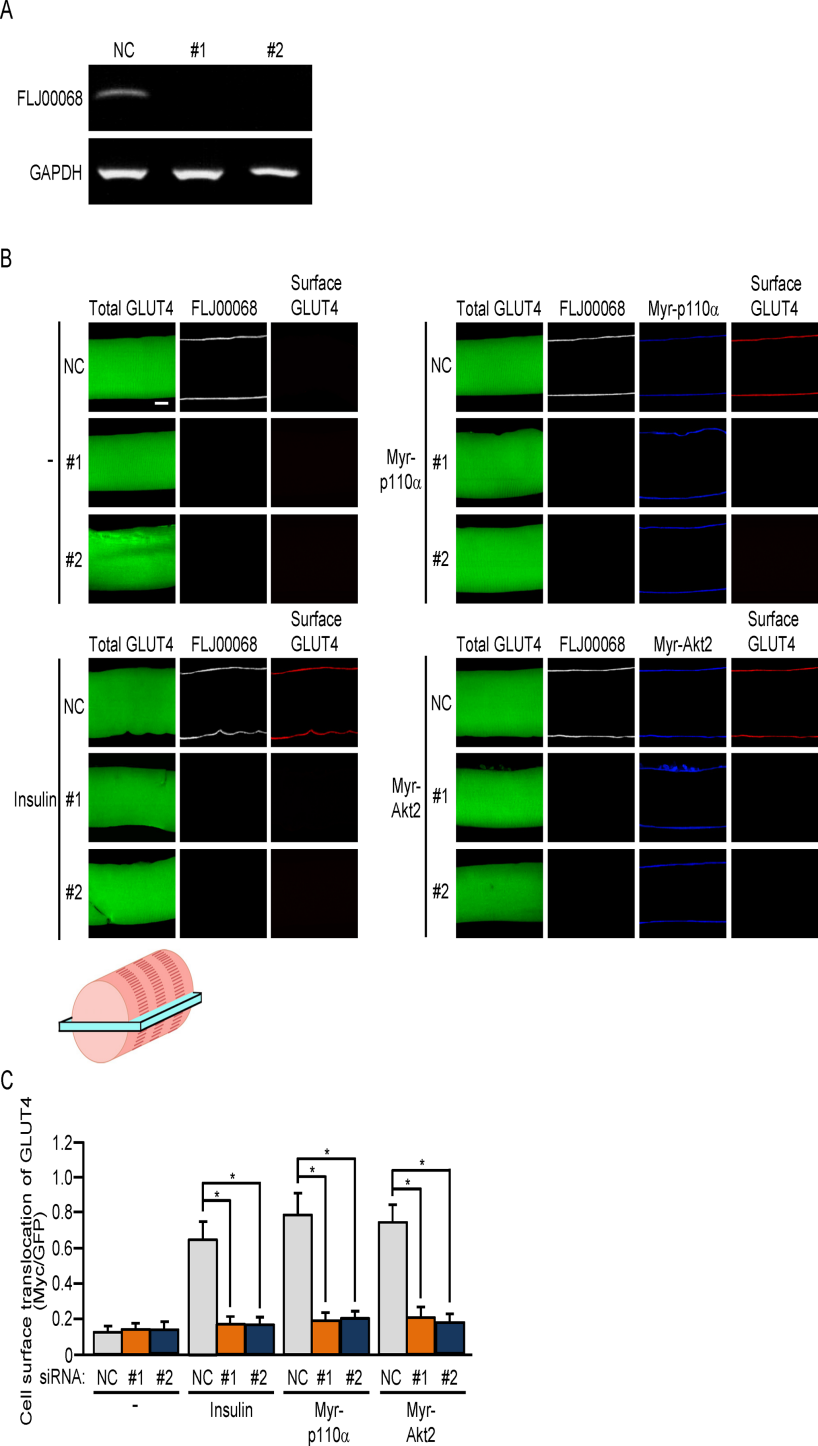
Inhibition of GLUT4 translocation to the sarcolemma by knockdown of FLJ00068. (A) Two siRNA duplexes against mouse FLJ00068 (#1 and #2) and a mixture of NC siRNA duplexes were introduced into gastrocnemius muscle fibers of wild-type mice. Expression levels of FLJ00068 and GAPDH were assessed by RT-PCR. (B) Expression vectors for GLUT4*myc*7-GFP, Myr-p110α, and Myr-Akt2, together with either one of two siRNA duplexes against mouse FLJ00068 (#1 and #2) and a mixture of NC siRNA duplexes, were introduced into gastrocnemius muscle fibers of wild-type mice. Insulin (175.5 μg/kg body weight) was administered intravenously. Endogenous FLJ00068 was detected by immunofluorescent staining with an anti-FLJ00068 antibody. Myr-p110α and Myr-Akt2 were detected by immunofluorescent staining with an anti-HA antibody. GLUT4*myc*7-GFP translocated to the sarcolemma was detected by immunofluorescent staining with an anti-Myc antibody. Scale bar, 20 μm. The position of the focal plane from which the image was acquired is shown at the bottom of the panel. (C) Translocation of GLUT4*myc*7-GFP to the sarcolemma shown in (B) was quantified. Gray, orange, and blue bars represent the treatment with NC, #1, and #2 siRNA duplexes, respectively. Data are shown as means ± S.E. (n = 6). *P < 0.001.

**Fig 3:**
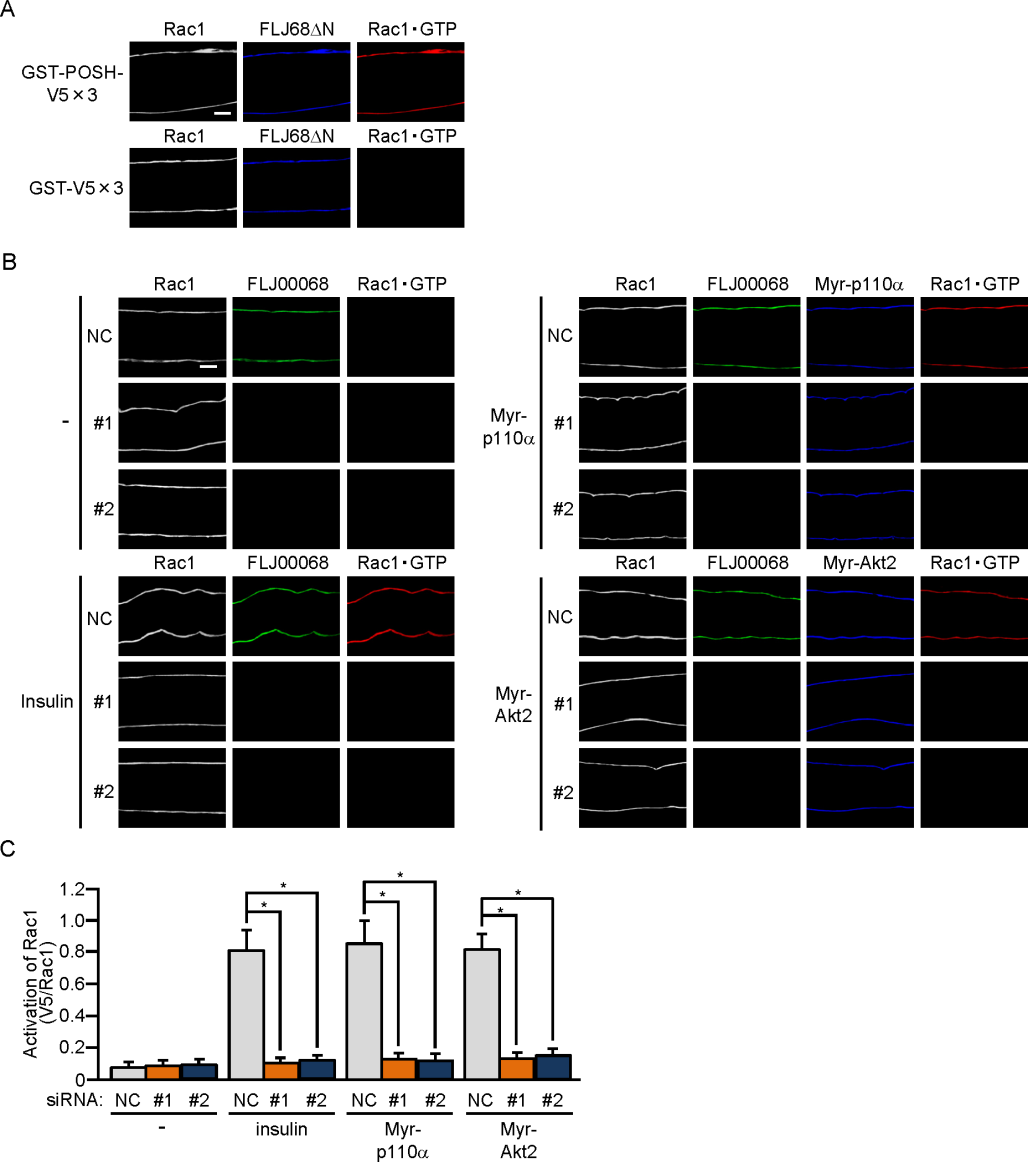
FLJ00068-dependent activation of Rac1 in mouse gastrocnemius muscle fibers. (A) The expression vector for FLJ68ΔN was introduced into gastrocnemius muscle fibers of wild-type mice. Endogenous Rac1 was detected by immunofluorescent staining with an anti-Rac1 antibody. FLJ68ΔN was detected by immunofluorescent staining with an anti-HA antibody. Activated Rac1 (Rac1·GTP) was visualized by immunofluorescent staining with an anti-V5 antibody after incubation with GST-POSH(251–489)-V5×3 or GST-V5×3. Scale bar, 20 μm. The position of the focal plane from which the image was acquired is shown in Fig 2B. (B) Expression vectors for Myr-p110α and Myr-Akt2, together with either one of two siRNA duplexes against mouse FLJ00068 (#1 and #2) and a mixture of NC siRNA duplexes, were introduced into gastrocnemius muscle fibers of wild-type mice. Insulin (175.5 μg/kg body weight) was administered intravenously. Endogenous Rac1 and FLJ00068 were detected by immunofluorescent staining with anti-Rac1 and anti-FLJ00068 antibodies, respectively. Myr-p110α and Myr-Akt2 were detected by immunofluorescent staining with an anti-HA antibody. Activated Rac1 (Rac1·GTP) was visualized by immunofluorescent staining with an anti-V5 antibody after incubation with GST-POSH(251–489)-V5×3. Scale bar, 20 μm. The position of the focal plane from which the image was acquired is shown in Fig 2B. (C) Activation of Rac1 shown in (B) was quantified. Gray, orange, and blue bars represent the treatment with NC, #1, and #2 siRNA duplexes, respectively. Data are shown as means ± S.E. (n = 6). *P < 0.001.

**Fig 4:**
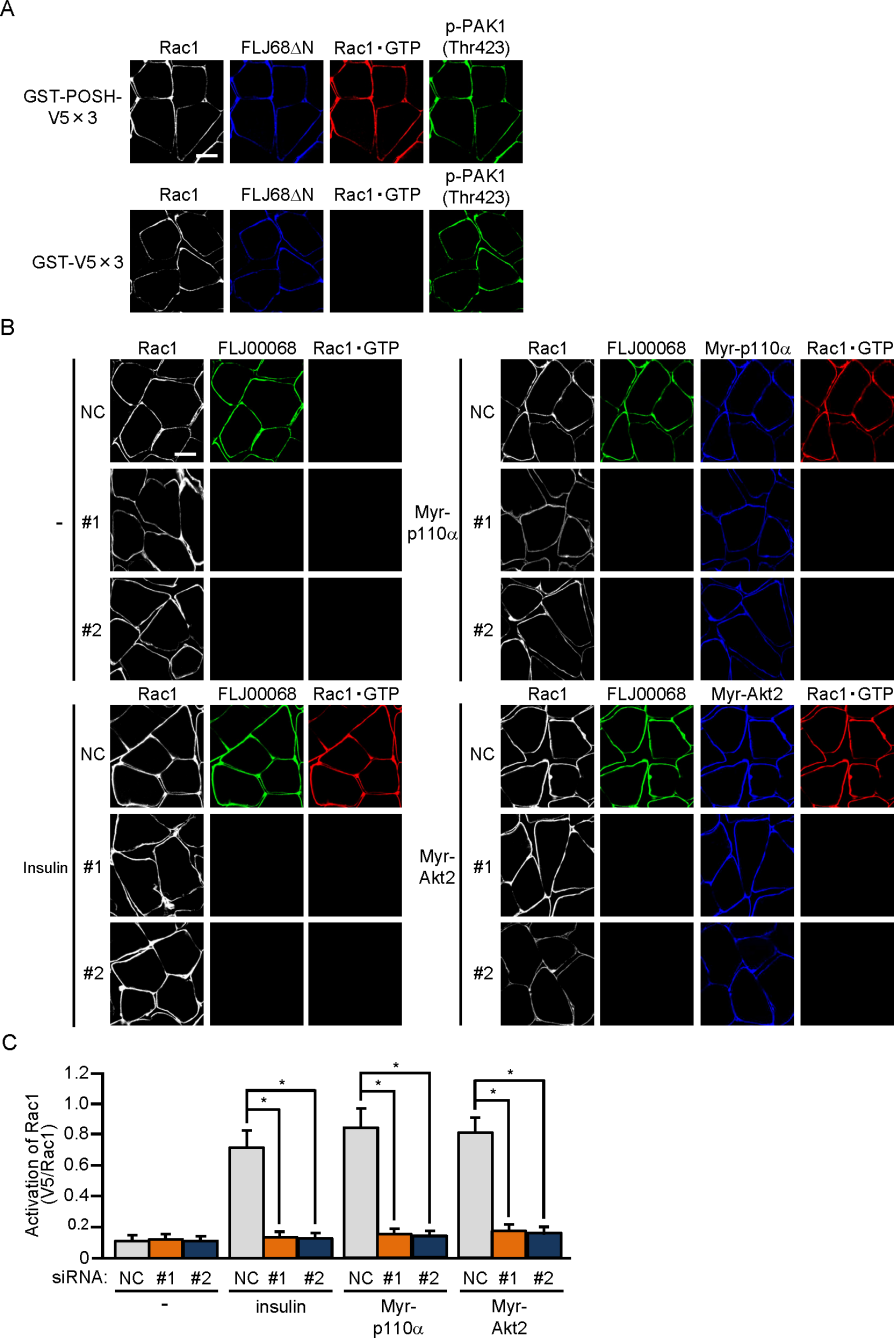
FLJ00068-dependent activation of Rac1 in mouse gastrocnemius muscle. (A) The expression vector for FLJ68ΔN was introduced into gastrocnemius muscle of wild-type mice. Endogenous Rac1 and phosphorylated PAK1 were detected by immunofluorescent staining with anti-Rac1 and anti-p-PAK1(Thr423) antibodies, respectively. FLJ68ΔN was detected by immunofluorescent staining with an anti-HA antibody. Activated Rac1 (Rac1·GTP) was visualized by immunofluorescent staining with an anti-V5 antibody after incubation with GST-POSH(251–489)-V5×3 or GST-V5×3. Scale bar, 20 μm. (B) Expression vectors for Myr-p110α and Myr-Akt2, together with either one of two siRNA duplexes against mouse FLJ00068 (#1 and #2) and a mixture of NC siRNA duplexes, were introduced into gastrocnemius muscle of wild-type mice. Insulin (175.5 μg/kg body weight) was administered intravenously. Endogenous Rac1 and FLJ00068 were detected by immunofluorescent staining with anti-Rac1 and anti-FLJ00068 antibodies, respectively. Myr-p110α and Myr-Akt2 were detected by immunofluorescent staining with an anti-HA antibody. Activated Rac1 (Rac1·GTP) was visualized by immunofluorescent staining with an anti-V5 antibody after incubation with GST-POSH(251–489)-V5×3. Scale bar, 20 μm. (C) Activation of Rac1 shown in (B) was quantified. Gray, orange, and blue bars represent the treatment with NC, #1, and #2 siRNA duplexes, respectively. Data are shown as means ± S.E. (n = 6). *P < 0.001.

As a first step to test the role of FLJ00068 in skeletal muscle insulin signaling, we confirmed the expression of this GEF in mouse gastrocnemius muscle. FLJ00068 mRNA expression was reported to be high in the human testis and pancreas [32]. In the adult mouse brain, FLJ00068 was highly expressed in the cerebellum, particularly in Purkinje neurons, as demonstrated by RT-PCR and immunohistochemical staining [33]. RT-PCR analysis revealed that the expression of FLJ00068 in mouse gastrocnemius muscle is comparable to that in the myoblast L6-GLUT4 cell line, in which a critical role of FLJ00068 in Akt2-mediated Rac1 activation has been shown [28], although lower than in the brain (Fig 1A). FLJ00068 is predominantly localized at the periphery of muscle fibers as demonstrated by immunohistochemical staining of the cross section of gastrocnemius muscle (Fig 1B). It should be noted that cytosolic and plasma membrane-localized FLJ00068 molecules cannot be distinguished by immunofluorescent microscopy of frozen sections due to the very thin cytoplasmic region in skeletal muscle fibers. Namely, it is not necessarily suggested that FLJ00068 is solely localized in the plasma membrane from the results shown in Fig 1B. The same is true for images of immunostained skeletal muscle fibers (see Figs 2–4).

It has been previously shown that constitutively activated mutants of PI3K (Myr-p110α) and Akt2 (Myr-Akt2), when ectopically expressed in mouse gastrocnemius muscle fibers, elicit GLUT4 translocation in a Rac1-dependent manner [24]. However, no experimental data that support the involvement of FLJ00068 in Myr-p110α- or Myr-Akt2-induced GLUT4 translocation in mouse gastrocnemius muscle have been presented, and thus, the effect of FLJ00068 knockdown on GLUT4 translocation to the sarcolemma was examined (Fig 2). For visualization of plasma membrane-localized GLUT4, we employed a GLUT4 reporter termed GLUT4*myc*7-GFP [31]. This GLUT4 reporter contains an exofacial Myc epitope tag, and can be detected by an anti-Myc antibody in non-permeabilized cells only when it is localized in the plasma membrane and establishes the proper topology. Two different siRNA duplexes against FLJ00068 (#1 and #2) were individually introduced together with the expression vector for the GLUT4 reporter GLUT4*myc*7-GFP into gastrocnemius muscle by electroporation, and the expression of FLJ00068 in GFP-positive muscle fibers was examined by RT-PCR 5 days after electroporation. As expected, the expression level of FLJ00068 was significantly reduced in GFP-positive muscle fibers (Fig 2A). Knockdown of FLJ00068 by the above siRNA molecules was also confirmed by immunofluorescent staining of isolated muscle fibers (Fig 2B). SiRNA knockdown of FLJ00068 markedly inhibited Myr-p110α- or Myr-Akt2-induced as well as insulin-induced GLUT4 translocation, suggesting a crucial role for FLJ00068 downstream of Akt2 (Fig 2B and 2C).

To further examine whether FLJ00068 is implicated in insulin signaling as an activator for Rac1, the activation state of Rac1 in gastrocnemius muscle was examined. Recently, we were successful in detecting the activated form of Rac1 in isolated muscle fibers by immunofluorescent microscopy by the use of a polypeptide probe that specifically recognizes activated Rac1 [25]. Specific interaction of the activation-specific Rac1 probe GST-POSH(251–489)-V5×3, but not the control polypeptide GST-V5×3, with GTP-bound activated Rac1, which were generated by ectopic expression of the constitutively activated FLJ00068 mutant FLJ68ΔN [21], was confirmed in gastrocnemius muscle fibers (Fig 3A). Rac1 activation following intravenous injection of insulin was remarkably inhibited by siRNA-mediated knockdown of FLJ00068 as revealed by the above immunofluorescent microscopy-based overlay assay (Fig 3B and 3C). Furthermore, Rac1 activation in response to ectopic expression of constitutively activated PI3K or Akt2 was highly sensitive to the inhibitory effect of FLJ00068 knockdown (Fig 3B and 3C). Taken together, these results strongly support the notion that FLJ00068 in fact acts as a specific Rac1 activator downstream of Akt2 in insulin signaling.

Although the above-described overlay assay for activated Rac1 allowed us to detect Rac1 activation in a single muscle fiber, it remains difficult to analyze multiple samples concomitantly because considerable efforts are made to prepare a large number of muscle fibers. In addition, distribution of activated Rac1 in a cross-section of muscle fibers cannot be visualized. To overcome these limitations, we attempted to detect Rac1 activation in frozen tissue sections by using the activation-specific Rac1 probe GST-POSH(251–489)-V5×3. Eventually, FLJ68ΔN-induced activation of Rac1 in a cross-section of gastrocnemius muscle was detected by this probe, but not by the control polypeptide GST-V5×3 (Fig 4A). Consistent with results obtained from paraformaldehyde-fixed muscle fibers, Rac1 activation occurred selectively at the periphery of muscle fibers. The protein kinase PAK1 is a target of activated Rac1 and the phosphorylation status of PAK1 has been considered to be virtually equivalent to the activation status of Rac1 [20]. Therefore, phosphorylated PAK1 in FLJ68ΔN-expressing gastrocnemius muscle was visualized by a phospho-specific antibody as a positive control experiment. We confirmed that phosphorylation of PAK1 actually occurred in both GST-POSH(251–489)-V5×3-treated and GST-V5×3-treated gastrocnemius muscle (Fig 4A).

The activation of Rac1 by insulin stimulation and constitutive activation of the possible upstream signaling components (i.e. PI3K and Akt2) was then assessed under similar conditions. Rac1 activation by insulin and constitutively activated PI3K and Akt2 was actually observed at the peripheral region of muscle fibers, and this Rac1 activation was almost completely inhibited in FLJ00068-knockdown muscle fibers (Fig 4B and 4C). Collectively, it is feasible that the GEF FLJ00068 plays a critical role for the activation of Rac1 downstream of Akt2 in skeletal muscle insulin signaling.

**Fig 5:**
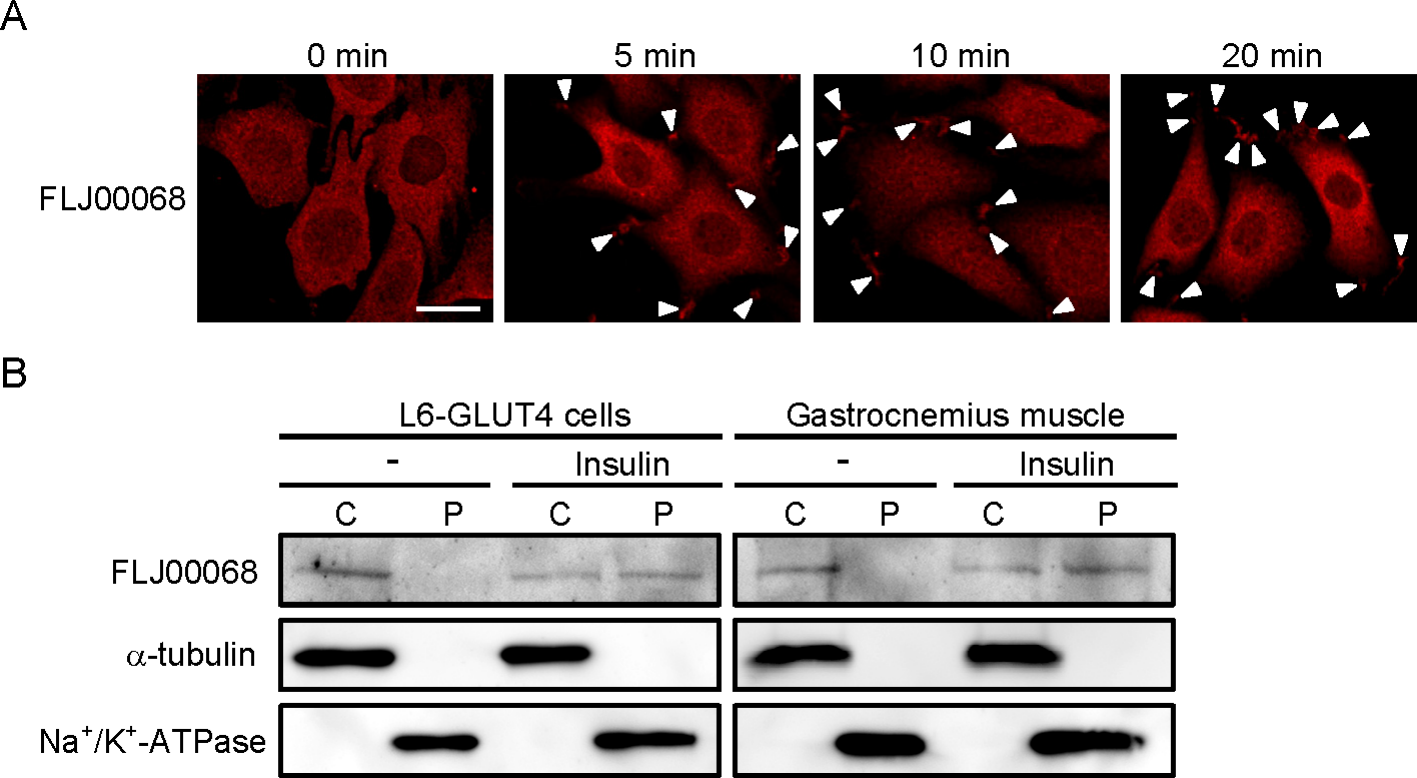
Insulin-dependent subcellular translocation of FLJ00068 to the plasma membrane in L6-GLUT4 cells and mouse gastrocnemius muscle. (A) Serum-starved L6-GLUT4 cells were stimulated with insulin for indicated times. Endogenous FLJ00068 was detected by immunofluorescent staining with an anti-FLJ00068 antibody. Arrow heads indicate the localization of FLJ00068 in the tip of membrane ruffle-like structures. Scale bar, 50 μm. (B) Localization of FLJ00068 in cytosol (C) and crude plasma membrane (P) fractions of insulin-stimulated and unstimulated L6-GLUT4 cells and mouse gastrocnemius muscle was examined by immunoblot analysis with an anti-FLJ00068 antibody. Α-tubulin and Na^+^/K^+^-ATPase were visualized as marker proteins for cytosol and crude plasma membrane fractions, respectively, by specific antibodies.

It is important to elucidate the molecular basis of insulin-dependent activation of FLJ00068, and therefore, as a first step, subcellular localization of FLJ00068 was examined. Translocation of FLJ00068 to the cell periphery, particularly to the tip of membrane ruffle-like structures, was observed within 5 minutes after the addition of insulin in L6-GLUT4 cells (Fig 5A). The peripheral localization of FLJ00068 was also observed after 20 minute stimulation and maintained at least for 60 minutes (Fig 5A and data not shown). The plasma membrane localization of FLJ00068 in insulin-stimulated L6-GLUT4 cells was also confirmed by subcellular fractionation and immunoblotting (Fig 5B). Similar results were obtained from subcellular fractionation of gastrocnemius muscle isolated from insulin-administered mice, supporting the notion that subcellular translocation of FLJ00068 to the plasma membrane in fact occurs following insulin stimulation in mouse skeletal muscle (Fig 5B). This change of subcellular localization of FLJ00068 is not obvious in images obtained by immunofluorescent microscopy due to the very thin cytoplasmic region in skeletal muscle fibers. (Figs 2B, 3B and 4B). Taken together, these findings may provide important clues to understand the regulatory mechanisms of FLJ00068 in insulin signaling.

## Discussion

In this study, we provide *in vivo* evidence that the GEF FLJ00068 regulates Rac1 downstream of Akt2, stimulating insulin-dependent GLUT4 translocation in mouse gastrocnemius muscle. Such a role for FLJ00068 had been revealed mainly by employing an L6 myoblast-derived cell line [21, 28], and thus *in vivo* evidence was eagerly awaited. Recently, considerable progress has been made in our laboratory in obtaining *in vivo* data for insulin signaling in mouse skeletal muscle. First, siRNA-mediated efficient knockdown of signaling molecules such as RalA in mouse skeletal muscle has been achieved [29]. Second, a novel technique to visualize the activation of small GTPases, such as Rac1 and RalA, *in situ* in skeletal muscle fibers has been developed [25, 28, 29]. Taking advantage of these newly established methodologies, we herein present *in vivo* data supporting the idea that FLJ00068 is actually required for Akt2 regulation of Rac1 in skeletal muscle. Involvement of Akt2 downstream of PI3K in the regulation of the FLJ00068-Rac1 pathway has also been verified by siRNA-based knockdown of Akt2 and treatment with a specific inhibitor against Akt in an L6 myoblast-derived cell line [23, 24]. However, the effect of Akt2 knockdown on PI3K-dependent Rac1 activation in skeletal muscle fibers has not been reported, and therefore we are now trying to perform such experiments.

We also report that immunofluorescent microscopy-based overlay assay for *in situ* detection of activated Rac1 by the use of an activation-specific probe is applicable, not only to paraformaldehyde-fixed isolated muscle fibers, but also to frozen sections of mouse skeletal muscle (Fig 4). It is noteworthy that, in principle, the activation state of Rac1 can be visualized in frozen sections of a variety of tissues besides skeletal muscle by this new approach. Moreover, activated forms of multiple signal transducing small GTPases may be counterstained in a tissue section. The utility of this novel method will be further demonstrated in future studies.

Given that FLJ00068 (puratrophin-1) is significantly expressed not only in skeletal muscle, but also in other tissues, such as the testis, pancreas, and brain [32, 33], it may also play a specific role in these tissues. The finding that a mutation in the human puratrophin-1 gene is associated with autosomal dominant spinocerebellar ataxia may imply its pivotal role in the brain [32]. Furthermore, the *Drosophila* ortholog of puratrophin-1 Pura has recently been implicated as a Rho1-GEF that is responsible for circadian oscillations of Rho1 activity in axons of s-LNv clock neurons, regulating their extension and retraction [34]. Pura expression is rhythmically regulated at a transcriptional level, peaking around dusk [34]. A circadian function of this GEF in mammals has also been suggested, based on its rhythmically regulated expression in the mouse pituitary [34, 35]. The role for FLJ00068 in the testis and pancreas remains obscure, and will be clarified in future studies.

It is important to reveal the molecular mechanisms by which Akt2 activates FLJ00068. As a first step, insulin-dependent subcellular translocation of FLJ00068 was examined in mouse skeletal muscle and L6-GLUT4 cells. Immunoblotting after subcellular fractionation as well as immunofluorescent microscopy revealed that FLJ00068 was translocated, although partly, to the plasma membrane in response to insulin stimulation (Fig 5). Upstream signal-dependent recruitment to the plasma membrane was actually reported to be a mechanism underlying the activation of several kinds of Dbl family GEFs, and thus may also be an activation mechanism for FLJ00068 [36–39]. Akt2-dependemt post-translational modifications and protein-protein interactions may be responsible for membrane translocation of FLJ00068, and may also relieve its GEF activity through conformational change. This model agrees well with experimental results that isolated tandem Dbl-homology and pleckstrin-homology domains exhibit constitutive activity [21]. Direct phosphorylation of FLJ00068 by Akt2, however, seems to be unlikely because no consensus sequence motif for Akt substrates has been found in FLJ00068, and rather, an unknown protein may mediate the Akt2 signal that causes the activation of FLJ00068. Detailed mechanisms whereby FLJ00068 is regulated in response to upstream insulin signals in skeletal muscle will be revealed in future.

Rac1 also participates in the regulation of contraction-dependent glucose uptake in skeletal muscle [40]. In fact, exercise and *ex vivo* muscle contraction lead to the activation of Rac1, whereas contraction-promoted glucose uptake was suppressed in m-*rac1*-KO mice [40]. In marked contrast to insulin signaling, contraction-stimulated glucose uptake is not mediated by PI3K [41], and therefore Rac1 activation in response to contraction is unlikely to be regulated by FLJ00068. Instead, another GEF for Rac1 may serve as a regulator in contraction-dependent signaling. Insulin enhances GLUT4-mediated glucose uptake in adipocytes as well as skeletal muscle. However, the role for Rac1 in glucose uptake signaling in adipocytes remains controversial. A previous report argues against the involvement of Rac1 in this signaling based on the observation that neither basal nor insulin-stimulated glucose uptake affected by the expression of a constitutively activated or dominant-negative Rac1 mutant in 3T3-L1 cells [42]. In contrast, a GEF termed P-Rex1 has been characterized to be a PI3K-dependent Rac1 regulator that mediates insulin-dependent actin cytoskeletal rearrangements and glucose uptake [43]. In fact, ectopically expressed P-Rex1 enhanced GLUT4 translocation in response to a low concentration of insulin, whereas knockdown of P-Rex1 partly reduced insulin-dependent glucose uptake [43]. In addition, the involvement of Rac1 in insulin signaling has been proposed in adipocytes, although the GEF that participates in the regulation of Rac1 in this signaling remains to be identified [44]. Therefore, Rac1 may play a crucial role in glucose uptake also in adipocytes, being regulated by several types of GEFs upon insulin stimulation. It is important to understand cell type- and signal-specific roles for Rac1-GEFs and their detailed regulatory mechanisms in insulin-dependent signal transduction.

In conclusion, the present study provides evidence that the GEF FLJ00068 acts as a direct Rac1 regulator downstream of Akt2, leading to translocation of GLUT4 to the sarcolemma in mouse gastrocnemius muscle.

## Abbreviations

GAPDH: glyceraldehyde-3-phosphate dehydrogenase
GEF: guanine nucleotide exchange factor
GFP: green fluorescent protein
GST: glutathione *S*-transferase
HA: hemagglutinin
m-*rac1*-KO: muscle-specific *rac1* knockout
Myr-Akt2: myristoylated Akt2
Myr-p110α: a myristoylated form of the phosphoinositide 3-kinase catalytic subunit p110α
NC: non-targeting control
PBS: phosphate-buffered saline
PI3K: phosphoinositide 3-kinase
RT-PCR: reverse transcription-polymerase chain reaction;
siRNA: small interfering RNA.
